# Systematic review: digital biomarkers of fatigue in chronic diseases

**DOI:** 10.1038/s41746-025-01939-x

**Published:** 2025-10-08

**Authors:** Nana Yaw Aboagye, Chloe Hinchliffe, Silvia Del Din, Wan-Fai Ng, Kenneth F. Baker, Mark R. Baker

**Affiliations:** 1 Translational and Clinical Research Institute, Faculty of Medical Sciences, Newcastle University, Newcastle upon Tyne, UK; 2 NIHR Newcastle Biomedical Research Centre, Newcastle upon Tyne Hospitals NHS Foundation Trust and Newcastle University, Newcastle upon Tyne, UK; 3 Department of Clinical Neurophysiology, Royal Victoria Infirmary, Newcastle upon Tyne, UK; 4 HRB Clinical Research Facility, University College Cork, Cork, Ireland; 5 Rheumatology Department, Newcastle upon Tyne Hospitals NHS Foundation Trust, Newcastle upon Tyne, UK

**Keywords:** Biomarkers, Health care, Biomedical engineering

## Abstract

This systematic review explores the relationship between digital biomarkers, measured using wearable devices, and fatigue in patients with chronic diseases. Studies included in this review focused on individuals with diseases or conditions in 13 broad categories: multiple sclerosis (MS); rheumatoid arthritis (RA); chronic obstructive pulmonary disease (COPD); long COVID; cancer; chronic fatigue syndrome (CFS); pulmonary sarcoidosis; Parkinson’s disease; chronic stroke; chronic inflammatory rheumatic disease (CIRD); Inflammatory Bowel Diseases (IBD), Primary Sjogren’s Syndrome (PSS), and Systemic Lupus Erythematosus (SLE). The review synthesizes findings on the correlation between objective digital biomarkers and self-reported fatigue, highlighting the potential for disease-specific digital biomarkers to inform personalized fatigue management. The results suggest that reduced physical activity, increased sedentary behavior and autonomic dysfunction are associated with fatigue levels across multiple disease conditions included in this review, though the strength of this association and the specific biomarkers involved vary across diseases.

## Introduction

Fatigue is one of the most prevalent and debilitating symptoms reported by individuals with chronic diseases, profoundly affecting their quality of life and daily functioning^1^. It is defined as extreme and persistent tiredness and reduced energy, which could be mental, physical, or both^2^. Fatigue in chronic diseases is multifaceted and often difficult to quantify and measure due to its subjective nature^3^. It varies greatly between individuals and across different diseases and disease states. In addition to reducing overall functional capacity, abnormal fatigue limits physical activity, disrupts daily routines, and exacerbates other symptoms such as pain and cognitive impairment, creating a vicious cycle that worsens patient health^4^.

In several chronic conditions, including multiple sclerosis (MS)^5^, rheumatoid arthritis (RA)^6^, chronic obstructive pulmonary disease (COPD)^7^, fatigue is a prevalent and disabling symptom. For example, in MS, fatigue affects between 75 and 90% of patients and is often reported as one of the most disabling symptoms^5,8^. In RA and COPD, fatigue is similarly widespread and contributes to reduced engagement in daily activities and diminished physical function^6,7^.

With the advent of wearable technologies and digital health devices, it has become increasingly feasible to objectively measure physical activity and physiological signals in individuals with chronic diseases^9^. Devices like accelerometers, fitness trackers, and ECG monitors can provide real-time data on step counts, movement intensity, heart rate and sedentary behavior^10^. Digital biomarkers encompass objective, quantifiable physiological and behavioral measurements acquired through these digital health devices^11^. The measurement of digital biomarkers is non-invasive, convenient, user-friendly, and suitable for home use, and thus ideal for longitudinal data collection.

**Fig. 1:**
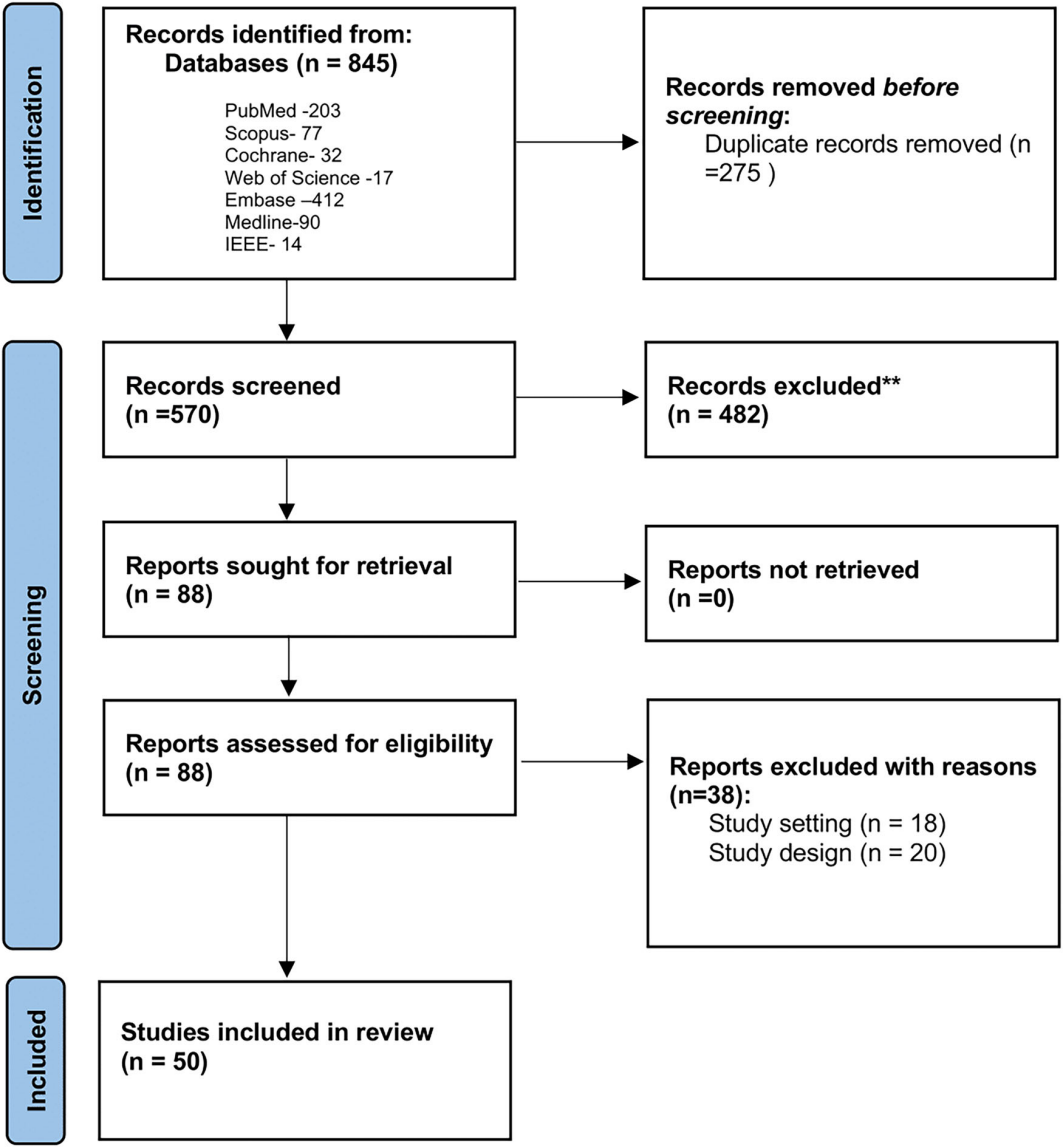
Prisma flowchart for the article selection process.

**Fig. 2:**
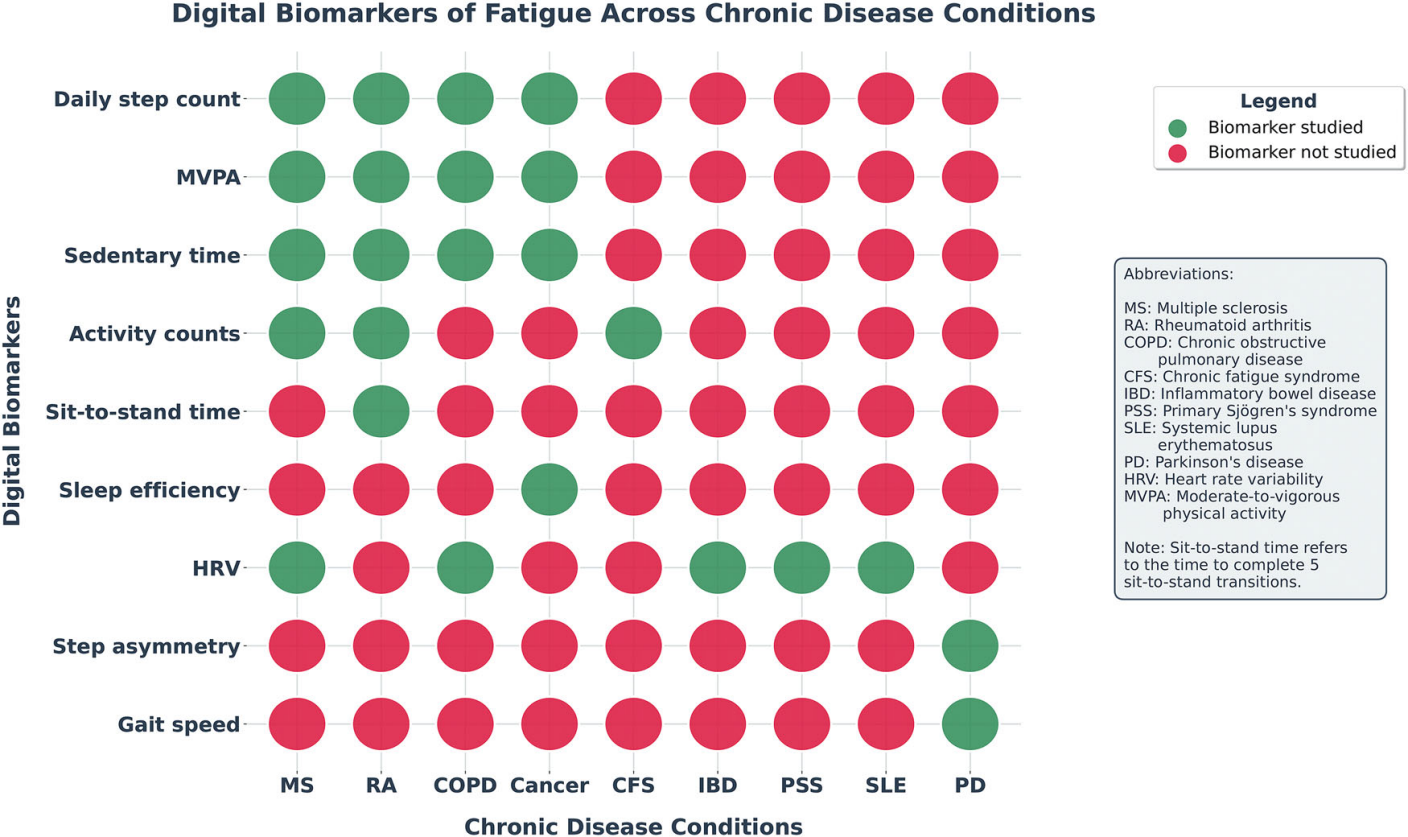
Summary of digital biomarkers across conditions. This figure offers a visual overview of the digital biomarkers examined in relation to fatigue across various chronic health conditions discussed in this review. Each row shows a specific digital biomarker (e.g., daily step count, HRV, sleep efficiency), while each column represents a different chronic condition. Green bubbles show that the biomarker has been studied in that condition, whereas red bubbles indicate it has not. This chart highlights both established research areas and gaps in current knowledge, providing insights into where future studies are needed to better understand the role of digital biomarkers in fatigue assessment and management. *Abbreviations:* MS, multiple sclerosis; RA, rheumatoid arthritis; COPD, chronic obstructive pulmonary disease; CFS, chronic fatigue syndrome; IBD, inflammatory bowel disease; PSS, primary Sjogren’s syndrome; SLE, systemic lupus erythematosus; PD, Parkinson’s disease; HRV, heart rate variability; MVPA, moderate-to-vigorous physical activity.

They are increasingly utilized across diverse areas of healthcare, where they are being incorporated into digital health systems for managing chronic conditions, neurological disorders, and mental health^12^. For instance, they are used to detect early cognitive decline in Alzheimer’s disease^13^. In mental health, speech and behavioral digital biomarkers are being explored to assess speech-based digital biomarkers, mood disorders and detect symptom changes over time^14,15^. These real-time, remote, and objective assessments allow for continuous monitoring, facilitate personalized care, and support timely clinical decisions.

Digital biomarkers thus offer an unprecedented opportunity to monitor fatigue in a way that complements self-reported fatigue measures. When combined, digital biomarkers and self-reported fatigue scores provide a holistic picture of how fatigue manifests and affects physical activity and physiology in chronic disease populations.

Recently, there has been growing interest in developing digital biomarkers of fatigue measured using smartphones and wearable devices in people with chronic diseases. This systematic review synthesizes current research examining the relationship between digital biomarkers and fatigue in individuals with chronic diseases. By focusing on studies that included wearable digital technologies, this review aims to provide insights into cross-disease fatigue patterns and explore how digital measures can inform personalized fatigue management strategies.

## Results

The search yielded 845 papers; after removing duplicates, 570 papers were screened by title and abstract. leaving 88 papers that met the inclusion criteria and were reviewed in full. After exclusions based on study setting, design, and full-text availability, the final sample comprised 50 articles. The flowchart illustrating the study selection process is presented in Fig. 1.

Of the 50 articles, the quality review identified 7 studies that were rated excellent, 41 studies were rated as good, and 2 studies were rated as moderate; all articles were included in this review (Supplementary Table 1). The review captured a total of 5573 participants (Supplementary Table 1). Participants came from 15 different chronic disease domains (Supplementary Table 1): Multiple Sclerosis (*n* = 27 studies, 2750 participants)^16–42^ Cancer patients and survivors (*n* = 3 studies, 1164 participants)^43–45^, Long Covid (*n* = 3 studies, 197 participants)^46–48^, COPD (*n* = 2 studies, 440 participants)^49,50^, Chronic Fatigue Syndrome (*n* = 2 studies, 101 participants)^51,52^, Rheumatoid Arthritis and Juvenile idiopathic Arthritis (*n* = 6 studies, 315 participants)^53–58^, Parkinson’s Disease (*n* = 2 studies, 60 participants)^57,59^, Chronic Stroke (*n* = 1 study, 57 participants)^60^, Chronic Inflammatory Rheumatic Disease (CIRD) (*n* = 1 study, 269 participants)^61^, Inflammatory Bowel Diseases (IBD) (*n* = 1 study, 18 participants)^57^, Primary Sjogren’s Syndrome (PSS) (*n* = 1 study, 18 participants)^57^, Huntington’s Disease (HD) (*n* = 1 study, 13 participants)^57^, Systemic Lupus Erythematosus (SLE) (*n* = 1 study, 16 participants)^57^, Pulmonary Sarcoidosis (*n* = 1 study, 15 participants)^58,62^.

Digital biomarkers consistently measured across studies included physical activity (PA), defined by time spent in various activity levels: vigorous physical activity (VPA), moderate physical activity (MPA), moderate-to-vigorous activity (MVPA), light physical activity (LPA), no physical activity (NPA), and sedentary activity (SA), based on pre-defined thresholds. Step count was the most reported measure (*n* = 9 studies), followed by time spent in MVPA, LPA, VPA, and MPA (*n* = 6 studies). For physiological measures, heart rate variability (HRV) and heart rate, typically derived from ECG or PPG sensors, were used in most studies (*n* = 8 studies). Sleep metrics, including total sleep time and Wake After Sleep Onset (WASO), were less commonly assessed (*n* = 3 studies), WASO, etc were measured in few studies (*n* = 3 studies).

Fourteen different fatigue scales were used in the included studies. The most common questionnaire used was the Fatigue Severity Scale (FSS), included in 13 studies^16,18,22,24,25,27,28,30,31,37,39,40,60,63^, followed by the Modified Fatigue Impact Scale (MFIS) (*n* = 6 studies)^23,29,33–35,64^ and Functional Assessment of Chronic Illness Therapy – Fatigue FACIT-F (*n* = 6 studies)^43–45,47,53,61^.

Torchio et al. found a significant negative correlation between time spent in MVPA and fatigue severity in 34 MS patients (*r* = −0.62, *p* < 0.001)^16^, while Blikman et al. observed that higher fatigue was linked to lower daily activity counts where fatigued MS patients had a higher percentage of their time sedentary and also spent less time in MVPA periods^17^. Other studies, such as that reported by Motl et al. identified a moderately predictive negative predictive relationship with physical activity, expressed as total movement counts, and fatigue^18^. Similarly, Grover et al. demonstrated a negative correlation between minutes spent in LPA and total fatigue. Moebus et al. reported using generalized additive models that increased heart rate and daily step counts related to fatigue in MS patients with a dysfunctional autonomic nervous system^20^. Gashi et al. confirmed significant correlations between step counts, HR, HRV, and fatigue scores^21^, while Jones et al. showed a link between fatigue symptoms and lower time spent in MVPA and daily steps^22^. Studies by Kratz et al., Eldemir et al., and Cederberg et al. also confirmed similar patterns of reduced activity in terms of activity counts per minute with higher fatigue^23–25^. Luostarinen et al. emphasized correlations between fatigue and physical activity metrics like steps per day and time spent in light to very vigorous activity^42^. Finally, Block et al. and Shema-Shiratzky et al. added further evidence on how physical activity metrics like daily step counts and time spent sedentary and fatigue are inversely related in MS patients^29,30^.

Hamy et al. found that RA patients with higher fatigue scores performed worse on functional tests, specifically taking longer to complete sit-to-stand time (the time to complete a set of 5 sit-to-stand transitions), with a significant correlation between sit-to-stand time and fatigue (*p* = 0.009)^53^. O’Brien et al. demonstrated that increased fatigue in RA patients was linked to more time spent sedentary and less time spent standing over six months^54^. Similarly, O’Leary et al. found a significant correlation between total time spent standing and fatigue in 76 RA patients^55^. Additionally, Armbrust et al. reported a negative correlation between fatigue and physical activity levels in the form of activity-related energy expenditure in children with juvenile idiopathic arthritis, with lower physical activity levels predicting higher fatigue (*r* = −0.30, *p* < 0.01)^56^.

Blondeel et al. reported that higher fatigue was significantly correlated with reduced levels of physical activity (amount and intensity), with average steps per day (as a measure of amount of PA) and average movement intensity during walking (as a measure of intensity of PA) (*r* = −0.21*, p* < 0.05)^50^.

Burton et al. found a weak correlation between time spent in physical activity measures such as LPA, MVPA, and VPA tracked by accelerometry, and fatigue in 82 long COVID patients, particularly in the afternoon and evening (−0.11 mean correlation)^46^. Haischer et al. observed that in 41 COVID-19 survivors, greater self-reported fatigue was significantly linked to less time in MVPA and fewer daily steps^47^.

Martin et al. found that in 87 cancer survivors, the fatigued group in the study, dichotomized based on a validated fatigue score cut-off point had a poorer sleep quality assessed by the amount of movement during the sleep period (mean sleep actigraphy and index of activity during sleep) compared to the non-fatigued group^43^. Sada et al. revealed that fatigued cancer survivors spent more time sitting or lying down, exhibited 52.2% less light activity and MVPA, and showed a significant positive correlation between fatigue scores and daily step count^44^. Vallance et al. demonstrated that, in 1049 breast cancer patients, more daily time spent in MVPA were linked to less fatigue, while more sedentary hours were associated with higher fatigue across multiple percentiles of fatigue scores^45^.

Vergauwen et al. found that CFS patients had significantly lower activity counts on weekdays compared to healthy controls, as revealed by a Mann–Whitney U test involving 66 CFS patients and 20 healthy controls^51^. Similarly, Evering et al. demonstrated that individuals with CFS were less physically active in the afternoon and evening, engaged in fewer high-intensity activities, and displayed more variability in their activity patterns expressed as mean acceleration per minute during the afternoon^52^.

Cho et al. found a significant negative correlation between fatigue scores and both time spent upright per day and time spent standing per day in a study involving 15 patients with pulmonary sarcoidosis^62^.

Pilotto et al. conducted a study of 42 Parkinson’s patients (21 with fatigue and 21 without), where unsupervised gait analysis using a MOVE IV device revealed that those with fatigue had significantly higher step time (*p* = 0.005) and step time asymmetry (*p* = 0.01) compared to those without fatigue, particularly during longer walking bouts^59^.

Antikainen et al. found statistically significant correlations between HRV measures (e.g., Beat to Beat interval (RR mean), Beat to Beat interval (RR max)) and both physical and mental fatigue in 46 participants with IBD, PSS and SLE^57^.

Rao et al. used machine learning models on Fitbit data from 269 participants with CIRDs, predicting physical and mental fatigue with moderate accuracy, with physical activity and resting heart rate being key predictors^61^.

Sanchez-Sanchez et al. conducted a study with 57 participants, finding a small but statistically significant negative correlation between fatigue scores and the percentage of time spent in LPA, based on accelerometry data collected over seven days^60^.

Among the digital biomarkers evaluated, physical activity metrics—including daily step count and moderate-to-vigorous physical activity (MVPA)—emerged as the most robust and consistently associated with patient-reported fatigue. These associations were observed across multiple chronic conditions, such as multiple sclerosis (MS), rheumatoid arthritis (RA), COPD, long COVID, and cancer, and were supported by several high-quality studies, resulting in high certainty of evidence (see Supplementary Table 3 for details).

Sedentary time also demonstrated a consistent positive association with fatigue across high-quality studies, achieving high certainty of evidence. Autonomic measures, particularly HRV, showed consistent negative associations with fatigue across multiple conditions, achieving high evidence certainty. In contrast, sleep-related measures and gait parameters were investigated in fewer studies and exhibited greater variability in both results and methodological quality. Sleep measures achieved low certainty of evidence due to inconsistent findings across only two studies, while gait parameters also received low certainty ratings as they were evaluated in only one study in Parkinson’s disease (Fig. 2).

## Discussion

This systematic review thoroughly explores the relationship between digital biomarkers, particularly physical activity, HRV, and fatigue across various chronic diseases. The results highlight several consistent patterns by which fatigue manifests and how digital biomarkers can offer objective insights into this subjective and often debilitating symptom. Although the disease context and specific biomarkers varied, common findings underscore the critical role digital measures could play in measuring, understanding, and managing fatigue.

The relationship between reduced physical activity and increased fatigue was consistent across most studies reviewed, regardless of disease type. In conditions such as multiple sclerosis (MS), rheumatoid arthritis (RA), COPD, cancer, and long COVID, lower levels of moderate-to-vigorous physical activity (MVPA), fewer daily steps, and prolonged sedentary behavior were strongly linked to higher fatigue levels. This trend was particularly evident in MS, as demonstrated by Torchio et al. and Blikman et al., where a higher burden of fatigue correlated with lower MVPA and fewer steps per day^16,17^. Blondeel et al. found a similar relationship in COPD patients, with those reporting higher fatigue showing significantly lower physical activity levels^50^. The role of physical activity in fatigue was also prominent in long COVID and cancer survivors. For example, Burton et al. found that long COVID patients who experienced greater fatigue exhibited reduced physical activity, especially in the afternoon and evening^46^. Similarly, in cancer survivors, Vallance et al. demonstrated that higher daily MVPA was associated with lower fatigue levels, suggesting that maintaining physical activity could mitigate the severity of fatigue in these populations^45^. These findings emphasize the value of digital biomarkers like MVPA and step count as objective, quantifiable indicators of fatigue. Wearable devices such as accelerometers and fitness trackers can provide continuous and real-time data on physical activity, offering a practical way to monitor fatigue and its fluctuations over time. Furthermore, the strong correlations between physical activity and fatigue across different conditions suggest that increasing physical activity may be a valuable intervention to alleviate fatigue as shown in a study conducted in women with primary Sjogren’s disease^65^. However, this would require further longitudinal studies to confirm causality.

Sedentary behavior, characterized by prolonged periods of sitting or lying down, was another important biomarker of fatigue across multiple chronic diseases. Several studies reported that fatigued patients spent significantly more time in sedentary activities and less time engaging in physical movement. This was particularly pronounced in MS and cancer patients. For example, Cederberg et al. found that fatigued MS patients were more sedentary and took fewer steps compared to non-fatigued individuals^25^. Sada et al. observed a similar trend in cancer survivors, where higher fatigue scores were associated with more time spent sitting or lying down, along with less moderate-to-vigorous physical activity (MVPA)^44^. These findings are notable as they suggest a possible bidirectional relationship between fatigue and sedentary behavior: while fatigue may contribute to increased sedentary time, a sedentary lifestyle could, in turn, intensify fatigue. While this may be a logical interpretation, caution is necessary to avoid over-interpretation, as causality cannot be conclusively inferred from these data. This relationship further raises important considerations for designing interventions for patients with chronic diseases, simply encouraging increased physical activity may not be sufficient if prolonged sedentary behavior is left unaddressed. Interventions that break up sedentary time, such as promoting LPA throughout the day, could be more effective in reducing fatigue, likely known as “activity pacing”, explored in some studies as a framework for managing chronic pain and fatigue^66^.

Gait parameters showed limited evidence as potential biomarkers of fatigue, with low certainty of evidence due to the restricted research base. Pilotto et al. demonstrated that Parkinson’s patients with higher fatigue exhibited longer step times and greater step time asymmetry compared to non-fatigued individuals, especially during longer walking bouts^59^. However, this finding is based on a single study with a small sample size (42 participants), limiting our ability to draw broader conclusions about gait parameters as fatigue biomarkers.

Significant limitations must be considered when interpreting gait-based fatigue measures in neurodegenerative diseases. Parkinson’s Disease is characterized by progressive gait impairment as the disease advances, making disease severity a potential confounding factor when applying gait analysis to assess fatigue^67,68^. The relationship between gait abnormalities and fatigue may be confounded by underlying motor dysfunction rather than representing a direct effect of fatigue on mobility. Additionally, the neurophysiological mechanisms of fatigue in progressive neurological disorders remain poorly characterized^68^, further complicating the interpretation of gait-fatigue relationships.

Given these limitations and the sparse evidence base consisting of only one study, more research is needed before gait measures can be reliably used as fatigue biomarkers in clinical practice. Future studies should include larger sample sizes, control for disease severity, and investigate gait-fatigue relationships across multiple neurodegenerative conditions to establish the generalizability of these findings.

In addition to physical activity, HRV emerged as a critical biomarker, particularly in diseases characterized by autonomic dysfunction, such as MS, immune-mediated inflammatory diseases (IMID), and neurodegenerative disorders. HRV metrics, such as the low-frequency to high-frequency (LF/HF) ratio and standard deviation of the normal-to-normal intervals (SDNN), have been shown to correlate with fatigue severity, suggesting that autonomic dysregulation plays a key role in fatigue. For example, Fei Yu et al. found that MS patients with higher fatigue exhibited an increased LF/HF ratio during the sit-to-stand test, indicating a disturbed balance between the sympathetic and parasympathetic components of the autonomic nervous system^28^. Similarly, Antikainen et al. reported significant correlations between some HRV metrics (e.g. beat to beat interval (RR) mean and maximum) and both physical and mental fatigue in IMID patients^57^. Lastly, Moebus et al. also reported that higher heart rate was associated with greater fatigue in MS patients with a dysfunctional autonomic nervous system^20^. These findings suggest that autonomic nervous system dysfunction may contribute to the experience of fatigue, making HRV a valuable digital biomarker for real-time monitoring of fatigue. Given the strong relationship between HRV and fatigue, HRV metrics could serve as a non-invasive and easily measurable indicator of fatigue severity. A study conducted in people with CFS who self-reported autonomic dysfunction using the Composite Autonomic Symptom Scale (COMPASS) revealed a similar pattern of symptoms of autonomic dysfunction strongly associated with reported fatigue^69^, with similar observations in Sjogren’s disease^70,71^. Wearable devices capable of measuring HRV could provide valuable insights into how fluctuations in autonomic function relate to daily variations in fatigue. This opens new possibilities for using HRV to personalize interventions that target both physical and autonomic aspects of fatigue.

Sleep-related measures showed limited and inconsistent evidence as fatigue biomarkers, achieving low certainty of evidence due to the sparse research base and mixed findings. Martin et al. found that cancer survivors with higher fatigue scores had poorer sleep quality as measured by actigraphy^43^. At the same time, Stephens et al. reported associations between sleep efficiency and fatigue in pediatric multiple sclerosis patients^26^. However, these findings come from only two studies with different populations and measurement approaches, limiting our ability to draw definitive conclusions about sleep-fatigue relationships.

The relationship between sleep and fatigue is complex and likely bidirectional, with evidence from both general population studies^72^ and chronic disease research^73^ supporting this relationship. Poor sleep potentially contributes to increased fatigue, while fatigue itself may disrupt sleep patterns. Large-scale community studies have demonstrated that higher fatigue levels are significantly associated with increased likelihood of sleep disturbances^72^. In contrast, studies in chronic fatigue syndrome have shown that disrupted sleep architecture and altered sleep stage transitions are characteristic features in patients experiencing persistent fatigue symptoms^73^. This bidirectional relationship makes it challenging to establish sleep parameters as reliable fatigue biomarkers without controlling for the underlying disease processes that may independently affect both sleep and fatigue. Additionally, the heterogeneity in sleep measurement approaches (actigraphy vs. sleep efficiency calculations) and the limited number of studies investigating sleep-fatigue relationships across different chronic diseases further reduce confidence in sleep as a universal fatigue biomarker. More longitudinal research is needed to clarify the causal relationships between sleep disturbances and fatigue and establish consistent methodological approaches for measuring sleep-related digital biomarkers across different chronic disease populations.

Our synthesis revealed a number of significant gaps in the current literature surrounding digital biomarkers of fatigue. Despite fatigue being a multifactorial experience, majority of the studies in this review focused on single biomarker categories in isolation. This fragmented approach neglects the potential of combining multiple digital signals, which might more accurately capture the complexity of fatigue. Future research should explore integrative models that blend different biomarker types, potentially yielding more powerful predictors of fatigue across contexts. Another limitation lies in the relative scarcity of longitudinal research. Only a small proportion of studies have employed designs that track biomarker changes over time in relation to fatigue. Without such temporal data, it’s difficult to fully understand how digital signals map onto the evolving experience of fatigue in daily life. More longitudinal studies are needed to capture this dynamic interplay. Furthermore, cross-condition validation remains notably underexplored. Very few studies have investigated the same biomarkers across multiple clinical populations using consistent protocols. This limits our ability to distinguish between general fatigue mechanisms and those specific to particular conditions. Studies designed for direct comparison across different pathologies could significantly advance this line of inquiry. While many investigations report correlations between digital biomarkers and fatigue, few go beyond that to assess diagnostic performance. Sensitivity, specificity, and clinically relevant cut-points are rarely discussed, limiting the practical utility of these tools in clinical decision-making. Establishing diagnostic thresholds and validating them in diverse populations will be a crucial next step. Another critical gap involves treatment response. There are not enough studies that have examined how digital biomarkers change in response to fatigue-targeted interventions. Understanding whether these markers are sensitive to treatment effects could position them as valuable outcome measures in clinical trials. Their ability to reflect change over time would enhance their role in evaluating intervention efficacy. The dominance of cross-sectional study designs also constrains our ability to infer causality. For example, while reduced physical activity is frequently associated with fatigue, it remains unclear whether it is a contributing factor, a consequence, or both. Experimental designs that manipulate contributing variables could help untangle such relationships and better establish causal pathways. Finally, methodological heterogeneity continues to be a major barrier to research synthesis. Studies vary widely regarding measurement tools, data processing algorithms, and outcome definitions. Without greater standardization in these areas, comparative analysis remains limited. A consensus on best practices would provide a solid foundation for future investigations and enhance the cumulative value of this growing body of work.

Despite the generally moderate-weak correlations observed between digital biomarkers and fatigue, these objective measures offer several compelling advantages for clinical practice. Rather than replacing self-reported outcomes, digital biomarkers are best positioned as complementary tools. Their objectivity may help identify discrepancies between patients’ subjective reports and actual physiological or behavioral changes that can be clinically meaningful. Another advantage lies in the capacity for continuous monitoring. Traditional clinical assessments provide only brief snapshots in time, often missing fluctuations that occur outside of appointments. Digital biomarkers, in contrast, can track fatigue in real-world environments over extended periods, potentially alerting clinicians to early signs of deterioration even before a patient becomes consciously aware of changes. Moreover, patterns in digital biomarkers may support more personalized fatigue interventions. For instance, if a patient’s data indicates consistently low physical activity levels, interventions might focus on gradual exercise programs. If sleep-related disruptions are prominent, the focus might shift to sleep hygiene and regulation strategies. Interestingly, studies using multivariate approaches have shown that combining multiple biomarkers can significantly improve the accuracy of fatigue prediction^64^. Where individual markers explain only modest proportions of variance, integrated models have demonstrated enhanced predictive power, suggesting that the future of fatigue monitoring lies in multi-dimensional digital phenotyping. Beyond the clinical and technical, patient engagement is another crucial consideration. Providing patients with visualizations of their own digital biomarker data, highlighting trends of improvement or decline, may empower them to take a more active role in managing their fatigue. This kind of biofeedback could enhance motivation, adherence, and overall self-efficacy. Nevertheless, several implementation challenges must be addressed before these tools can be widely adopted. These include ensuring cost-effectiveness, overcoming technical and usability barriers, safeguarding data privacy, and promoting acceptance among both patients and providers. These practical concerns should be addressed in tandem with continued validation efforts to ensure that digital biomarkers of fatigue are not only scientifically robust but also clinically and ethically sound.

One of the key strengths of this systematic review is its comprehensive coverage of multiple chronic diseases, including multiple sclerosis (MS), rheumatoid arthritis (RA), COPD, long COVID, cancer, Parkinson’s disease, and more. This broad scope allows for a comparison of fatigue patterns across diverse conditions. It highlights commonalities in the relationship between fatigue and digital biomarkers, such as physical activity and HRV. Another strength is the focus on real-world, objective data collection through wearable devices, which provide continuous and non-invasive monitoring of patients’ activity, HRV, and sleep. This overcomes the inherent limitations of self-reported fatigue assessments, which are often subjective and can vary due to recall bias. Wearable technologies, like accelerometers and fitness trackers, offer quantifiable data that can be collected over extended periods, providing a more holistic view of fatigue dynamics and give better insights into biomarkers with potential clinical utility compared to e.g., gait lab/laboratory measurements. The review also highlights the potential of digital biomarkers to facilitate personalized fatigue management. By integrating objective measures into routine clinical care, there is an opportunity to tailor interventions and monitor the effectiveness of treatments in real-time, improving patient outcomes. This is especially important for chronic diseases where fatigue is a pervasive and debilitating symptom.

Despite its strengths, this review has several limitations. The predominance of observational studies limits the ability to establish causality between digital biomarkers (e.g., physical activity, HRV) and fatigue. Without RCTs or intervention studies, it remains unclear whether increasing activity or improving sleep quality directly reduces fatigue or if these relationships are merely correlative. Another limitation is the variability in assessment tools and digital biomarkers. Fatigue was measured using different scales (e.g., FSS, FACIT-F), introducing heterogeneity that complicates comparisons. Similarly, variations in wearable devices (e.g., ActiGraph, Fitbit) may lead to inconsistencies in physical activity and HRV measurements. Additionally, most studies lacked long-term data, despite fatigue being a dynamic symptom that fluctuates over time. Longitudinal studies are needed to capture the full impact of fatigue and assess whether changes in activity, HRV, or sleep led to lasting improvements. Further considerations include the diversity of participants, limiting generalizability across ethnic and geographic groups, and potential seasonal variations, which might impact results, especially in regions further from the equator. In summary, this systematic review identified physical activity metrics (particularly step count and MVPA) as the most robust digital biomarkers of fatigue across chronic conditions, with moderate to strong correlations consistently observed across disease contexts. The certainty of evidence varied considerably across biomarker types, with physical activity metrics achieving the highest confidence ratings. As technology advances, digital biomarkers may play an increasingly important role in objectively capturing the multifaceted nature of fatigue, ultimately improving assessment and management of this debilitating symptom across chronic disease populations.

## Methods

This systematic review was conducted in accordance with the Preferred Reporting Items for Systematic Reviews and Meta-Analyses (PRISMA) guidelines^74^. The protocol was prospectively registered with PROSPERO (registration number: CRD42024498838). The completed PRISMA checklist is provided in the Supplementary Table 4. Following the PICO framework, our research question was: In adults with chronic diseases (Population), which digital biomarkers measured through wearable and connected technologies (Intervention/Exposure) are associated with or can predict subjective fatigue measures (Outcome)?

Nine databases were searched from the earliest records to March 2024: PubMed, Scopus, Cochrane, Web of Science, CINAHL, PsycINFO, Embase, MEDLINE, and IEEE. The reporting of this systematic review was guided by the standards of the Preferred Reporting Items for Systematic Review and Meta-Analysis (PRISMA) guidelines (Supplementary Table 4)^74^. The search strategy was developed in PubMed using medical subject headings (MeSH) based on the key concepts of ‘digital biomarkers’, ‘chronic diseases’, and ‘fatigue’ (Supplementary Table 2). Duplicates were removed from the compiled articles, and the titles and abstracts were then reviewed independently by two reviewers.

Inclusion criteria ensured reviewed studies had participants with at least one chronic disease and used wearable sensors in a real-world environment to measure digital biomarkers. Studies were excluded if they monitored participants under controlled conditions (such as a laboratory or hospital), participants were healthy individuals, or if they explored physiological fatigue or fatigue induced by exercise. Articles were excluded if written in a language other than English, as well as conference abstracts, case reports, literature reviews, meta-analysis, grey literature, and study protocols.

Two reviewers (NA and CH) independently extracted data using a standardized form. Key data included the study setting, clinical population, type and model of sensor, fatigue questionnaire used, sensor measures derived, and study results. An independent reviewer (MB) resolved any discrepancies in screening or extraction.

Quality appraisal was performed using the Downs and Black Quality Appraisal form^75^. Two reviewers (NA and CH) completed assessments independently, and an average quality score was derived. Certainty of evidence for each biomarker-fatigue association was systematically determined through a multi-factorial approach that considered: (1) the methodological quality of supporting studies, (2) consistency of findings across studies, (3) the total number of studies investigating each biomarker, and (4) the heterogeneity of populations studied. Certainty was rated as “high” when a biomarker demonstrated consistent findings across three or more high-quality studies. “Moderate” certainty was assigned when consistency was present, but supporting studies were of moderate quality. “Low” certainty was assigned when fewer than three studies investigated the biomarker or when findings were inconsistent, regardless of individual study quality.

We acknowledge that the certainty of evidence ratings was not specified in the original PROSPERO protocol but were added during the review process to enhance the robustness of our evidence synthesis.

## Supplementary information

Supplementary Materials

## Data Availability

No datasets were generated or analysed during the current study.

## References

[1] Saligan, L. N. et al. The biology of cancer-related fatigue: a review of the literature. *Support Care Cancer* **23**, 2461–2478 (2015). 25975676 10.1007/s00520-015-2763-0 PMC4484308

[2] Hernandez-Ronquillo, L., Moien-Afshari, F., Knox, K., Britz, J. & Tellez-Zenteno, J. F. How to measure fatigue in epilepsy? The validation of three scales for clinical use. *Epilepsy Res.* **95**, 119–129 (2011). 21482076 10.1016/j.eplepsyres.2011.03.010

[3] Davies, K., Dures, E. & Ng, W.-F. Fatigue in inflammatory rheumatic diseases: current knowledge and areas for future research. *Nat. Rev. Rheumatol.* **17**, 651–664 (2021). 34599320 10.1038/s41584-021-00692-1

[4] Rowe, P. C. et al. Myalgic encephalomyelitis/chronic fatigue syndrome diagnosis and management in young people: a primer. *Front. Pediatr.* **5**, 121 (2017). 28674681 10.3389/fped.2017.00121 PMC5474682

[5] Krupp, L. B. Fatigue in multiple sclerosis. *CNS Drugs* **17**, 225–234 (2003). 12665396 10.2165/00023210-200317040-00002

[6] Dar, W. R., Mir, I. A., Siddiq, S., Nadeem, M. & Singh, G. The assessment of fatigue in rheumatoid arthritis patients and its impact on their quality of life. *Clin. Pract.* **12**, 591–598 (2022). 35892448 10.3390/clinpract12040062 PMC9332162

[7] Goertz, Y. M. J. et al. Fatigue in patients with chronic obstructive pulmonary disease: protocol of the Dutch multicentre, longitudinal, observational FAntasTIGUE study. *BMJ Open* **8**, e021745 (2018). 29643168 10.1136/bmjopen-2018-021745 PMC5898336

[8] Freal, J. E., Kraft, G. H. & Coryell, J. K. Symptomatic fatigue in multiple sclerosis. *Arch. Phys. Med Rehabil.* **65**, 135–138 (1984). 6703889

[9] Huhn, S. et al. The impact of wearable technologies in health research: scoping review. *JMIR Mhealth Uhealth* **10**, e34384 (2022). 35076409 10.2196/34384 PMC8826148

[10] Roos, L. G. & Slavich, G. M. Wearable technologies for health research: opportunities, limitations, and practical and conceptual considerations. *Brain Behav. Immun.* **113**, 444–452 (2023). 37557962 10.1016/j.bbi.2023.08.008 PMC11233111

[11] Babrak, L. M. et al. Traditional and digital biomarkers: two worlds apart?. *Digit. Biomark.* **3**, 92–102 (2019). 32095769 10.1159/000502000 PMC7015353

[12] Coravos, A., Khozin, S. & Mandl, K. D. Developing and adopting safe and effective digital biomarkers to improve patient outcomes. *NPJ Digit. Med.* **2**, 14, 10.1038/s41746-019-0090-4 (2019). 30868107 10.1038/s41746-019-0090-4 PMC6411051

[13] Gold, M. et al. Digital technologies as biomarkers, clinical outcomes assessment, and recruitment tools in Alzheimer’s disease clinical trials. *Alzheimer’s Dement. Transl. Res. Clin. Interv.* **4**, 234–242, 10.1016/j.trci.2018.04.003 (2018). 10.1016/j.trci.2018.04.003 PMC6021547 29955666

[14] Robin, J. et al. Evaluation of speech-based digital biomarkers: review and recommendations. *Digit. Biomark.* **4**, 99–108, 10.1159/000510820 (2020). 33251474 10.1159/000510820 PMC7670321

[15] Jacobson, N. C., Weingarden, H. & Wilhelm, S. Digital biomarkers of mood disorders and symptom change. *NPJ Digital Med.* **2**, 3. 10.1038/s41746-019-0078-0 (2019). 10.1038/s41746-019-0078-0 PMC6550284 31304353

[16] Torchio, A. et al. Objective and subjective measures of daily physical activity in persons with Multiple Sclerosis beginning a rehabilitation regime: a cross-sectional study. *Mult. Scler. Relat. Disord.* **68**, 104394 (2022). 36544306 10.1016/j.msard.2022.104394

[17] Blikman, L. J. et al. Is physical behavior affected in fatigued persons with multiple sclerosis?. *Arch. Phys. Med. Rehabil.* **96**, 24–29 (2015). 25239283 10.1016/j.apmr.2014.08.023

[18] Motl, R. W. & McAuley, E. Symptom cluster as a predictor of physical activity in multiple sclerosis: preliminary evidence. *J. Pain. Symptom. Manag.* **38**, 270–280 (2009). 10.1016/j.jpainsymman.2008.08.004 19329276

[19] Grover, S. A. et al. Physical activity and its correlates in youth with multiple sclerosis. *J. Pediatr.* **179**, 197–203.e2 (2016). 27717498 10.1016/j.jpeds.2016.08.104

[20] Moebus, M. et al. Meaningful digital biomarkers derived from wearable sensors to predict daily fatigue in multiple sclerosis patients and healthy controls. *iScience* **27**, 108965 (2024). 38362266 10.1016/j.isci.2024.108965 PMC10867654

[21] Gashi, S. et al. Modeling multiple sclerosis using mobile and wearable sensor data. *npj Digit Med.* **7**, 64 (2024). 38467710 10.1038/s41746-024-01025-8 PMC10928076

[22] Jones, C. D. et al. Do fatigue and depression have a bivariate association with device-measured physical activity behavior in persons with multiple sclerosis?. *Disabil. Rehabil.* **46**, 2522–2527 (2024). 37350026 10.1080/09638288.2023.2225876

[23] Kratz, A. L. et al. Daily temporal associations between physical activity and symptoms in multiple sclerosis. *Ann. Behav. Med.* **53**, 98–108 (2019). 29697757 10.1093/abm/kay018 PMC6301314

[24] Eldemir, K. et al. Associations between fatigue and physical behavior in patients with multiple sclerosis with no or minimal disability. *Fatigue Biomed. Health Behav.* **9**, 69–78 (2021).

[25] Cederberg, K. L. J. et al. Physical activity and sedentary behavior timing in fatigued and nonfatigued adults with multiple sclerosis. *Arch. Phys. Med. Rehabil.* **103**, 1758–1765 (2022). 35063422 10.1016/j.apmr.2021.12.022 PMC9294061

[26] Motl, R. W. et al. Physical activity and quality of life in multiple sclerosis: intermediary roles of disability, fatigue, mood, pain, self-efficacy and social support. *Psychol. Health Med.* **14**, 111–124 (2009). 19085318 10.1080/13548500802241902 PMC2893350

[27] Abbadessa, G. et al. Assessment of multiple sclerosis disability progression using a wearable biosensor: a pilot study. *J. Clin. Med* **10**, 1160 (2021). 33802029 10.3390/jcm10061160 PMC8001885

[28] Yu, F. et al. A wireless body measurement system to study fatigue in multiple sclerosis. *Physiol. Meas.* **33**, 2033–2048 (2012). 23151461 10.1088/0967-3334/33/12/2033

[29] Block, V. J. et al. Continuous daily assessment of multiple sclerosis disability using remote step count monitoring. *J. Neurol.* **264**, 316–326 (2017). 27896433 10.1007/s00415-016-8334-6 PMC5292081

[30] Shema-Shiratzky, S. et al. A wearable sensor identifies alterations in community ambulation in multiple sclerosis: contributors to real-world gait quality and physical activity. *J. Neurol.* **267**, 1912–1921 (2020). 32166481 10.1007/s00415-020-09759-7

[31] Gervasoni, E. et al. Physical activity in non-disabled people with early multiple sclerosis: a multicenter cross-sectional study. *Mult. Scler. Relat. Disord.* **64**, 103941 (2022). 35691235 10.1016/j.msard.2022.103941

[32] Sagawa, Y. et al. Physical activity during weekdays and weekends in persons with multiple sclerosis. *Sensors* **21**, 1170–1182 (2021). 34067409 10.3390/s21113617 PMC8197006

[33] Kasser, S. L. et al. Symptom variability, affect and physical activity in ambulatory persons with multiple sclerosis: understanding patterns and time-bound relationships. *Disabil. Health J.* **10**, 207–213 (2017). 27814947 10.1016/j.dhjo.2016.10.006

[34] VanDyk, T. et al. Digital phenotypes of instability and fatigue derived from daily standing transitions in persons with multiple sclerosis. *IEEE Trans. Neural Syst. Rehabil. Eng.* **31**, 2279–2286 (2023). 37115839 10.1109/TNSRE.2023.3271601 PMC10408384

[35] Hilty, M. et al. Continuous monitoring with wearables in multiple sclerosis reveals an association of cardiac autonomic dysfunction with disease severity. *Mult. Scler. J. Exp. Transl. Clin.* **8**, 20552173221103436 (2022). 35677598 10.1177/20552173221103436 PMC9168869

[36] Stuart, C. M. et al. Physical activity monitoring to assess disability progression in multiple sclerosis. *Mult. Scler. J. Exp. Transl. Clin.* **6**, 2055217320975185 (2020). 33343919 10.1177/2055217320975185 PMC7727071

[37] Ng, A. V. & Kent-Braun, J. A. Quantitation of lower physical activity in persons with multiple sclerosis. *Med. Sci. Sports Exerc.* **29**, 517–523 (1997). 9107635 10.1097/00005768-199704000-00014

[38] Braakhuis, H. E. M. et al. Three distinct physical behavior types in fatigued patients with multiple sclerosis. *J. Neuroeng. Rehabil.* **16**, 105 (2019). 31443714 10.1186/s12984-019-0573-1 PMC6708224

[39] Motl, R. W. & McAuley, E. Pathways between physical activity and quality of life in adults with multiple sclerosis. *Health Psychol.* **28**, 682–689 (2009). 19916636 10.1037/a0015985

[40] Neal, W. N. et al. Is symptomatic fatigue associated with physical activity and sedentary behaviors among persons with multiple sclerosis?. *Neurorehabil. Neural Repair* **34**, 505–511 (2020). 32340521 10.1177/1545968320916159 PMC8796123

[41] Stephens, S. et al. Sleep, physical activity, and psychological outcomes in children and adolescents with pediatric onset multiple sclerosis. *Mult. Scler. Relat. Disord.* **79**, 105025 (2023). 37776826 10.1016/j.msard.2023.105025

[42] Luostarinen, M. et al. Correlation of fatigue with disability and accelerometer-measured daily physical activity in patients with relapsing-remitting MS. *Mult. Scler. Relat. Disord.* **78**, 104908 (2023). 37517311 10.1016/j.msard.2023.104908

[43] Martin, T. et al. The relationship between fatigue and actigraphy-derived sleep and rest-activity patterns in cancer survivors. *Curr. Oncol.* **28**, 1170–1182 (2021). 33802111 10.3390/curroncol28020113 PMC8025824

[44] Sada, Y. H. et al. Harnessing digital health to objectively assess cancer-related fatigue: the impact of fatigue on mobility performance. *PLoS ONE* **16**, e0246101 (2021). 33636720 10.1371/journal.pone.0246101 PMC7910036

[45] Vallance, J. K. et al. Associations of device-measured physical activity and sedentary time with quality of life and fatigue in newly diagnosed breast cancer patients: baseline results from the AMBER cohort study. *Cancer* **129**, 296–306 (2023). 36367438 10.1002/cncr.34531 PMC10695099

[46] Burton, C. et al. Within and between-day variation and associations of symptoms in Long Covid: Intensive longitudinal study. *PLoS ONE* **18**, e0280343 (2023). 36656830 10.1371/journal.pone.0280343 PMC9851560

[47] Haischer, M. H. et al. Heart rate variability is reduced in COVID-19 survivors and associated with physical activity and fatigue. *Physiol. Rep.* **12**, e15912 (2024). 38243329 10.14814/phy2.15912 PMC10799199

[48] Burton, C., Dawes, H., Goodwill, S., Thelwell, M. & Dalton, C. Symptom variation, correlations, and relationship to physical activity in Long Covid: intensive longitudinal study. https://www.medrxiv.org/content/10.1101/2022.05.31.22275746v1 (2022).

[49] Driver, C. N., Novotny, P. J. & Benzo, R. P. Differences in sedentary time, light physical activity, and steps associated with better COPD quality of life. *Chronic Obstr. Pulm. Dis.* **9**, 34–44 (2022). 34783232 10.15326/jcopdf.2021.0230 PMC8893964

[50] Blondeel, A. et al. Factors associated to physical activity in patients with COPD: an ecological approach. *Respir. Med.* **219**, 107424 (2023). 37820971 10.1016/j.rmed.2023.107424

[51] Vergauwen, K. et al. An exploratory study of discrepancies between objective and subjective measurement of the physical activity level in female patients with chronic fatigue syndrome. *J. Psychosom. Res* **144**, 110417 (2021). 33773330 10.1016/j.jpsychores.2021.110417

[52] Evering, R. M., Tönis, T. M. & Vollenbroek-Hutten, M. M. Deviations in daily physical activity patterns in patients with the chronic fatigue syndrome: a case control study. *J. Psychosom. Res.* **71**, 129–135 (2011). 21843746 10.1016/j.jpsychores.2011.04.004

[53] Hamy, V. et al. Patient-centric assessment of rheumatoid arthritis using a smartwatch and bespoke mobile app in a clinical setting. *Sci. Rep.* **13**, 18311 (2023). 37880288 10.1038/s41598-023-45387-7 PMC10600111

[54] O’Brien, C. M. et al. Pain and fatigue are longitudinally and bi-directionally associated with more sedentary time and less standing time in rheumatoid arthritis. *Rheumatology* **60**, 4548–4557 (2021). 33493311 10.1093/rheumatology/keab029 PMC8487306

[55] O’Leary, H. et al. Relationship between pain and sedentary behavior in rheumatoid arthritis patients: a cross-sectional study. *Arthritis Care Res.* **73**, 990–997 (2021). 10.1002/acr.24207 32277738

[56] Armbrust, W. et al. Fatigue in patients with Juvenile Idiopathic Arthritis: relationship to perceived health, physical health, self-efficacy, and participation. *Pediatr. Rheumatol.* **14**, 65 (2016). 10.1186/s12969-016-0125-1 PMC5139083 27919265

[57] Antikainen, E. et al. Assessing fatigue and sleep in chronic diseases using physiological signals from wearables: a pilot study. *Front. Physiol* **13**, 968185 (2022). 36452041 10.3389/fphys.2022.968185 PMC9702812

[58] Pinto, A. J. et al. Increased prolonged sitting in patients with rheumatoid arthritis during the COVID-19 pandemic: a within-subjects, accelerometer-basedstudy. *Int. J. Environ. Res. Public Health* **20**, 3944 (2023). 36900955 10.3390/ijerph20053944 PMC10001724

[59] Pilotto, A. et al. Unsupervised but not supervised gait parameters are related to fatigue in Parkinsonas disease: a pilot study. *Front. Aging Neurosci* **15**, 1279722 (2023). 38076532 10.3389/fnagi.2023.1279722 PMC10702762

[60] Sánchez-Sánchez, M. L. et al. Association of barriers, fear of falling and fatigue with objectively measured physical activity and sedentary behavior in chronic stroke. *J. Clin. Med.* **10**, 1170–1182 (2021). 33806818 10.3390/jcm10061320 PMC8005010

[61] Rao, C. et al. Association of digital measures and self-reported fatigue: a remote observational study in healthy participants and participants with chronic inflammatory rheumatic disease. *Front. Digit. Health* **5**, 1099456 (2023). 37426890 10.3389/fdgth.2023.1099456 PMC10324580

[62] Cho, P. S. P. et al. Physical inactivity in pulmonary sarcoidosis. *Lung* **197**, 285–293 (2019). 30888492 10.1007/s00408-019-00215-6 PMC6520325

[63] Motl, R. W. et al. Energy cost of walking and its association with gait parameters, daily activity, and fatigue in persons with mild multiple sclerosis. *Neurorehabil. Neural Repair* **26**, 1015–1021 (2012). 22466791 10.1177/1545968312437943

[64] Chikersal, P. et al. Predicting multiple sclerosis outcomes during the COVID-19 stay-at-home period: observational study using passively sensed behaviors and digital phenotyping. *JMIR Ment. Health* **9**, e38495 (2022). 35849686 10.2196/38495 PMC9407162

[65] Strömbeck, B. et al. Physical capacity in women with primary Sjögren’s syndrome: a controlled study. *Arthritis Rheum.* **49**, 681–688 (2003). 14558054 10.1002/art.11384

[66] Antcliff, D. et al. Testing a newly developed activity pacing framework for chronic pain/fatigue: a feasibility study. *BMJ Open* **11**, e045398 (2021). 34880007 10.1136/bmjopen-2020-045398 PMC8655535

[67] Sharafkhah, M., Moayedi, F., Alimi, N., Haghighi Fini, Z. & Massoudifar, A. Motor and non-motor predictors of freezing of gait in Parkinson’s disease: a retrospective cohort study. *J. Bodyw. Mov. Ther.* **40**, 1774–1781, 10.1016/j.jbmt.2024.10.043 (2024). 39593523 10.1016/j.jbmt.2024.10.043

[68] Zwarts, M. J., Bleijenberg, G. & van Engelen, B. G. Clinical neurophysiology of fatigue. *Clin. Neurophysiol.* **119**, 2–10, 10.1016/j.clinph.2007.09.126 (2008). 18039594 10.1016/j.clinph.2007.09.126

[69] Newton, J. L. et al. Symptoms of autonomic dysfunction in chronic fatigue syndrome. *QJM* **100**, 519–526 (2007). 17617647 10.1093/qjmed/hcm057

[70] Newton, J. L. et al. Autonomic symptoms are common and are associated with overall symptom burden and disease activity in primary Sjögren’s syndrome. *Ann. Rheum. Dis.* **71**, 1973 (2012). 22562982 10.1136/annrheumdis-2011-201009 PMC3488764

[71] Ng, W. F. et al. Primary Sjögren’s syndrome is associated with impaired autonomic response to orthostasis and sympathetic failure. *QJM* **105**, 1191–1199 (2012). 22976617 10.1093/qjmed/hcs172 PMC3508582

[72] Hyun, M. K. How fatigue level is related to sleep disturbances: a large cross-sectional community study. *Eur. J. Integr. Med.* **49**, 102097, 10.1016/j.eujim.2021.102097 (2022).

[73] Kishi, A., Struzik, Z. R., Natelson, B. H., Togo, F. & Yamamoto, Y. Dynamics of sleep stage transitions in healthy humans and patients with chronic fatigue syndrome. *Am. J. Physiol. Regul. Integr. Comp. Physiol.* **294**, R1980–R1987, 10.1152/ajpregu.00925.2007 (2008). 18417644 10.1152/ajpregu.00925.2007 PMC9741833

[74] Page, M. J. et al. The PRISMA 2020 statement: an updated guideline for reporting systematic reviews. *BMJ* **372**, n71 (2021). 33782057 10.1136/bmj.n71 PMC8005924

[75] Downs, S. H. & Black, N. The feasibility of creating a checklist for the assessment of the methodological quality both of randomised and non-randomised studies of health care interventions. *J. Epidemiol. Community Health* **52**, 377 (1998). 9764259 10.1136/jech.52.6.377 PMC1756728

