# Supplementary material for: Systematic review: digital biomarkers of fatigue in chronic diseases
Source: NPJ Digit Med. 2025 Oct 8;8:602. doi: 10.1038/s41746-025-01939-x (PMC12508163; doi:10.1038/s41746-025-01939-x)
Supplement: Supplementary file 1 — Supplementary Materials [file 41746_2025_1939_MOESM1_ESM.pdf]

Supplementary Materials

Supplementary Table 1| Summary of all studies used in this review.

**Author**	**Study title**	**Sample size**	**Sensor name and Monitoring duration**	**Digital Biomarkers measured**	**Fatigue measures/scale**	**Results**
Alessandro Torchio (2022) [13]	Objective and subjective measures of daily activity in person with multiple sclerosis beginning a rehabilitation regime	34 participants (21 female, 13 male)<br><br>Mean age: 52 years	Fitbit Vasa<br><br>1 week	Daily steps, minutes of physical activity, MVPA	Fatigue Severity Scale (FSS)	Moderate correlation between FSS and MVPA; fatigue negatively correlated with MVPA
Elisa Grevasoni (2022) [48]	Physical activity in non-disabled people with early multiple sclerosis: A multicenter cross-sectional study	58 MS participants, 20 healthy subjects<br><br>Mean age: 39 years	Wrist GENE Activ accelerometer<br><br>7 days	Activity levels (NPA, LPA, MPA, VPA)	Fatigue Severity Scale (FSS)	Longer rest periods for fatigued MS with EDSS 0-1.5
Christopher Burton (2023) [35]	Long covid symptoms and physical activity	82 adults with long COVID (69 analyzed)<br><br>Mean age: 50 years	Axivity AX3<br><br>14 days	Sedentary, light, moderate, vigorous activity	VAS input for fatigue	Weak association between activity and fatigue (-0.11 correlation)

Robert W. Motl (2009) [15]	Symptoms cluster as a predictor of physical activity in multiple sclerosis	292 MS participants<br><br>Mean age: 48 years	ActiGraph accelerometer<br><br>7 days	Physical activity levels	Fatigue Severity Scale (FSS)	Fatigue, depression, and physical activity cluster negatively related to activity behavior
Astrid Blondeel (2023) [34]	Factors associated with physical activity in patients with COPD	148 patients with COPD<br><br>Mean age: 68 years	Dynaport Move Monitor<br><br>7 days	Average steps, movement intensity	CIS subjective fatigue subscale	Higher fatigue associated with lower activity intensity
Lyan J. Blikman (2015) [14]	Is Physical behaviour affected in fatigued persons with multiple sclerosis	23 fatigued persons with MS	ActiGraph GT3X-accelerometer<br>7 days	Physical activity counts per day, intensity	CIS fatigue domain questionnaire	Higher fatigue associated with lower activity intensity
Richard M.H. Evering (2011) [42]	Deviations in daily physical activity patterns in patients with chronic fatigue syndrome: A case control study	Patients diagnosed with CFS.<br><br>Age range: 18-65 years	Tri-axial piezoelectric accelerometer (X Sens)<br><br>7 days	Activity patterns, intensity levels	Not specified	CFS patients less physically active in the afternoon and evening
Max Moebus (2024) [17]	Meaningful digital biomarker derived from wearable sensors to predict daily fatigue in multiple sclerosis patients and healthy controls	27 MS patients, 23 healthy controls<br><br>Mean age: 34-39 years	Everion Biofourmis AG<br><br>2 weeks	Heart rate, skin temperature, step count	VAS fatigue questionnaire	Heart rate variability linked to fatigue

Stephanie A. Grover (2016) [16]	Physical and it correlates in youth with Multiple sclerosis	68 patients (27 MS, 41 mono-ADS) and 31 healthy controls.<br><br>Mean age: 15 years	ActiGraph 7164 acceleromet er<br>7 days	Light, moderate, vigorous minutes of physical activity	Varni pediatric quality of life inventory fatigue scale	Negative correlation between light physical activity and total fatigue
Kuni Vergauwen (2021) [41]	An exploratory study of discrepancies between objective and subjective measurement of the physical activity level in female patients with chronic fatigue syndrome	66 female patients with CFS, 20 healthy controls.<br><br>Age range: 20-62 yearsz	Actical acceleromet er<br>6 consecutive days	Activity counts (AC)	CFS symptom list	CFS patients have lower activity counts than healthy controls
Yoshimasa Sagawa (2021) [49]	Physical activity during weekdays and weekends in persons with multiple sclerosis	41 MS participants, 16 healthy controls	ActiGraph WGT3xH<br>7 days	Physical activity, sedentary time	Fatigue Impact Scale (FIS)	No statistically significant correlation between fatigue and physical activity
Valentin Hamy (2023) [30]	Patient-centric assessments of rheumatoid arthritis using a smartwatch and bespoke mobile app in a clinical setting	28 RA patients, 28 age-matched controls.<br><br>Mean age:58 years	Apple Watch Series 4<br><br>14 days	Activity counts (AC)	FACIT-F questionnair e	Higher fatigue scores associated with worse sit-to-stand transition time
Shkurta Gashi (2024) [18]	Modeling multiple sclerosis using mobile and wearable sensors	55 patients with MS, 24 healthy controls.	Biovotion Everion armband<br><br>2 weeks	Heart rate, skin temperature, step count	VAS Fatigue Questionnair e	Heart rate variability linked to fatigue

		Mean age:35 years				
Rakesh Malhotra (2021) [51]	Physical activity in hemodialysis patients on non-dialysis and dialysis days	45 participants with end-stage kidney disease.<br><br>Mean age:61 years.	Fitbit Charge 2 tracker<br><br>4 weeks	Steps per day	PROMIS short form fatigue questionnaire	No correlation between daily steps and fatigue
Andrea Pilotto (2023) [44]	Unsupervised but not supervised gait parameters are related to fatigue in Parkinson's diseases: a pilot study	42 Parkinson's disease patients.	MOVE IV inertial sensor.<br><br>4 consecutive days	Gait parameters per walking bouts	PD Fatigue Scale	Higher step time and step time asymmetry in fatigued patients
Emmi Antikainen (2022) [46]	Assessing fatigue and sleep in chronic diseases using physiology signals from wearables	31 patients with neurodegenerative disorders, 66 with immune diseases.<br>Mean age:36-80 years.	Vital patch wearable<br>5 consecutive days	ECG, heart rate, sleep	Likert fatigue scale	Correlation between R-R interval variability and fatigue
Charito Rao (2023) [45]	Association of digital measures and self-reported fatigue: a remote observational study in healthy participants and participants with chronic inflammatory rheumatic disease	269 participants with CIR, 105 healthy volunteers.<br><br>Mean ag:45 years	Fitbit wearable<br><br>1 month	Physical activity, sedentary time, heart rate	FACIT-fatigue questionnaire	Machine learning model accuracy of 75.2% in predicting fatigue

Tyler VanDyk (2023) [52]	Digital phenotypes of instability and fatigue derived from daily standing transitions in persons with multiple sclerosis	23 pwMS<br><br>Mean age:50 years	Biostamp npoint sensors<br><br>6 weeks	Activity transitions, standing and sitting	Modified Fatigue Impact Scale (MFIS)	Activity transitions predict symptoms such as fatigue
C. Danielle Jones (2024) [19]	Do fatigue and depression have a bivariate association with device-measured physical activity in persons with multiple sclerosis	210 pwMS<br><br>Mean age:50 years	ActiGraph model GT3X+<br><br>7 days	Physical activity, sedentary behavior	FSS	Fatigue associated with less physical activity
Anna L. Kratz (2019) [20]	Daily temporal association between physical activity and symptoms in multiple sclerosis	107 pwMS<br><br>Mean age:50 years	PRO-Diary wrist monitor<br><br>7 days	Activity counts per minute	Modified Fatigue Impact Scale	Negative association between activity and fatigue at certain times
Tristan Martin (2021) [38]	Relationship between fatigue and actigraphy-derived sleep and rest activity patterns in cancer survivors	87 cancer survivors<br><br>Mean age:55 years	MotionWatch 8<br><br>14 days	Sleep patterns, activity levels	FACIT-F	Fatigue associated with less physical activity
Hellen O'Leary (2021) [32]	Relationship between pain and sedentary behavior in rheumatoid arthritis patients	76 RA patients<br><br>Age range:18-80 years.	activPAL 4 accelerometer<br><br>7 days	Sedentary behavior and physical activity	VAS Fatigue	Correlation between sedentary time and pain

Jeff K. Vallance (2023) [40]	Associations of device-measured physical activities and sedentary time with quality of life and fatigue in newly diagnosed breast cancer	1049 breast cancer patients<br><br>Age range:18-80 years.	activPAL and ActiGraph GT3x+<br>7 days	Physical activity and sedentary time	FACIT-F	Increased MVPA associated with less fatigue
J. Nicholas Brenton [73]	Six-minute walk as a measure of walking capacity and endurance in patients with pediatric-onset multiple sclerosis	45 POMS patients, 85 controls<br><br>Mean age:15 years	ActiGraph GTX sensor<br><br>7 days	Walking capacity, physical activity	MFIS	No association between activity levels and fatigue
Kader Eldemir (2021) [21]	Association between fatigue and physical behavior in patients with multiple sclerosis with minimal disability	40 MS patients, 30 controls<br><br>Mean age:38 years	ActiGraph GT3x+<br><br>3 days	Physical activity, counts per day	FSS and FIS	Fatigue correlated with sedentary behavior
Mare Hilty (2022) [53]	Continuous monitoring with wearables in multiple sclerosis reveals an association of cardiac autonomic dysfunction with disease severity	55 pwMS, 24 controls<br><br>Mean age:35 years	Everion Biosensor<br><br>2 weeks	Heart rate, physical activity	FSMC	No significant correlation between HRV and fatigue
Charlotte M. Stuart (2020) [54]	Physical activity monitoring to assess disability progression in multiple sclerosis	56 people with progressive MS<br><br>Mean age:53 years	SenseWear armband	Activity counts, energy expenditure	Chalder Fatigue Scale	No significant correlation between fatigue and activity levels

			6 days every 6 months for 2.5 years			
Peter S. P. Cho (2019) [43]	Physical inactivity in pulmonary sarcoidosis	15 sarcoidosis patients, 14 controls<br><br>Mean age:50 years	ActivPAL<br><br>6 days	Step count, sit-to-stand transitions	FAS	More fatigue linked to increased sedentary behavior
Robert W. Motl (2012) [25]	Energy cost of walking in its association with gait parameters, daily activity, and fatigue in persons with multiple sclerosis	34 MS patients	ActiGraph 7164<br><br>7 days	Energy expenditure, gait parameters	FSS	Higher energy expenditure associated with fatigue
Michael H. Haischer (2024) [36]	Heart rate variability is reduced in COVID-19 survivors and associated with physical activity and fatigue	41 COVID-19 survivors, 41 matched controls Age range:19-60 years.	ActiGraph GT3X<br><br>7 days	Heart rate variability and physical activity	FACIT-F	Lower HRV associated with more fatigue
N. G. Alexander V. (1997) [55]	Quantification of lower physical activity in persons with multiple sclerosis	17 MS patients<br><br>Mean age:46 years	Tri-Trac-R3D<br><br>7 days	Physical activity intensity	FSS	No significant correlation between activity and fatigue
H.E.M Braakhuis (2019) [56]	Three distinct physical behavior types in fatigued patients with multiple sclerosis	212 MS participants<br><br>Mean age:48 years	ActiGraph GT3X+<br><br>7 days	Physical activity patterns	Fatigue subscale of the Checklist Individual Strength (CIS20r)	Fatigued patients show distinct activity patterns

Wineke Armbrust (2016) [33]	Fatigue in patients with juvenile idiopathic arthritis: relationship to perceived health, physical health self-efficacy, and participation	80 JIA patients<br><br>Age range:8-13 years	Actical accelerometer<br><br>7 days	Activity counts, energy expenditure	PedsQL-Fatigue	Fatigue negatively correlated with physical activity
Robert W. Motl (2009) [57]	Pathways between physical activity and quality of life in adults with multiple sclerosis	276 MS participants<br><br>Mean age:48 years	ActiGraph accelerometer<br><br>6 months	Physical activity, fatigue, quality of life	FSS	Physical activity influences fatigue and QoL
V. J. Block (2017) [28]	Continuous daily assessment of multiple sclerosis disability using remote step count monitoring	99 RRMS patients<br><br>Mean age: 50 years.	Fitbit Inspire HR<br><br>4 weeks	Step count, daily activity	MFIS	Daily step count negatively correlated with fatigue
Shirley Shema-Shiratzky (2020) [29]	A wearable sensor identifies alterations in community ambulation in multiple sclerosis	44 MS patients, 60 healthy controls<br><br>Mean age:50 years	AX3 sensor<br><br>7 days	Activity type, intensity	FSS	Activity type correlates with fatigue
C. Noelle Driver (2022) [58]	Differences in sedentary time and physical activity in patients with COPD	292 COPD patients<br><br>Mean age: 68 years	SenseWear Pro armband<br><br>7 days	Physical activity, sedentary behavior	Fatigue and QoL scale	Fatigue linked to less MVPA
Gianmarco Abbadessa (2021)	Assessment of multiple sclerosis disability	25 MS patients	Samsung Gear S2	Daily steps, physical activity	FSS	No correlation between fatigue and steps

[26]	progression using wearable biosensors	Mean age:40 years	30 days			
Prerna Chikersal (2022) [59]	Predicting multiple sclerosis outcomes using digital phenotyping during COVID-19	56 MS participants<br><br>Mean age:43 years	Fitbit Inspire HR<br><br>12 weeks	Steps, heart rate, sleep, location	MFIS	Digital measures predicted fatigue with 75.2% accuracy
Katie L. J. Cederberg (2022) [22]	Physical activity and sedentary behavior timing in fatigued and non-fatigued adults with multiple sclerosis	218 pwMS<br><br>Mean age:59 years	ActiGraph GT3X<br><br>7 days	Physical activity, sedentary time	Fatigue Severity Scale (FSS)	Fatigued participants had less MVPA and more sedentary time
Yvone H. Sada (2021) [39]	Harnessing digital health to assess cancer-related fatigue: the impact of fatigue on mobility performance	28 cancer survivors<br><br>Mean age:66 years	PAM sensor<br><br>48 hours	Mobility performance, activity levels	FACIT-F	Fatigue associated with decreased mobility performance
Fei Yu (2012) [27]	A wireless body measurement system to study fatigue in multiple sclerosis	17 MS participants<br><br>Mean age:46 years	Wearable body measuremen t system<br><br>24 hours	Heart rate variability, physical activity	FSS	Fatigue correlated with heart rate variability
Susan L. Kasser (2017) ** [60]	Symptoms variability and physical activity in	4 participants with MS	ActiGraph acceleromet er	Daily physical activity counts	Modified Fatigue Impact Scale	No co-variation between physical activity and fatigue scores

	ambulatory persons with multiple sclerosis	Mean age:45 years	28 days			
Ciara M. O'Brien (2021) [31]	Pain and fatigue longitudinally related to sedentary time in rheumatoid arthritis	54 RA participants<br><br>Mean age:59 years	ActivPAL 3<br><br>7 days	Sedentary behavior and physical activity	Multidimensional Fatigue Scale	Pain and fatigue related to increased sedentary time
Whitney N. Neal (2020) [61]	Is Symptomatic Fatigue Associated With Physical Activity and Sedentary Behaviors Among Persons With Multiple Sclerosis?	252 pwMS<br><br>Mean age:59 years	ActiGraph GT3X+<br><br>7 days	Physical activity, sedentary time	FSS	Fatigue associated with more sedentary time and less MVPA
M. Luz Sánchez-Sánchez (2021) [47]	Association of Barriers, Fear of Falling and Fatigue with Objectively Measured Physical Activity and Sedentary Behavior in Chronic Stroke	57 participants<br><br>Mean age:58 years	Actigraph GT3X+<br><br>7 days	Physical activity, sedentary time	Fatigue Severity Scale (FSS)	Small but statistically significant negative correlation was found between fatigue scores and the percentage of time spent in light physical activity (LPA), based on accelerometry data collected over seven days
C. Noelle Driver (2022) [62]	Differences. Incidentally, time like physical activity and steps associated with better COPD quality of life.	292 patients<br><br>Mean age:68 years	SenseWear Pro armband<br><br>7 days	Physical activity, sedentary behavior	Chronic Respiratory Disease Questionnaire(fatigue subscale)	Multiple linear regression using physical activity as dependent variable revealed that individuals with 1-pont better fatigue averaged 579(351-814) more steps per a day.
S. Stephens et al. (2023) [63]	Sleep, physical activity, and psychological outcomes in children	Children with POMS and age-matched controls	Actigraph (GT3X) accelerometer and	Sleep quality metrics: total sleep time, number of	PedsQL-MFS scale (PEDSQL Multidimensi	Sleep efficiency was associated with less sleep/rest fatigue (Spearman Rho = 0.44, p = 0.03). More minutes awake after sleep

	and adolescents with pediatric onset multiple sclerosis.	Mean age:15 years	Actiwatch (Phillips Respironics)<br><br>10 days	awakenings, wake after sleep onset (WASO) and accelerometer was used to collect PA data including time spent in SED, light (LPA), and moderate to vigorous (MVPA)	onal Fatigue Scale)	onset were associated with worse fatigue (Spearman Rho = 0.6, p < 0.01).
Marco Luostarinen (2023) [64]	Correlation of fatigue with disability and accelerometer-measured daily physical activity in patients with relapsing-remitting MS	41 adults with relapsing-remitting MS(RRMS).<br><br>Mean age:41 years	Actigraph WGT3x<br><br>7 days	Physical activities comprising sedentary (SED, light (LPA)moderate, vigorous and very vigorous physical activity as MVPS, LPA, SED, and step count per day	MFIS and FSS questionnair e	After Pearson correlation analyses on activity measures and fatigue score, total activity (MVPS) daily steps correlated with fatigue in RRMS groups as well as in the EDSS-low group but not in the other groups. Sit-up correlated t fatigue only in the EDSS- high group as measured with the MFIS.
Robert W. Motl (2009) [65]	Physical activity of life in multiple sclerosis: intermediary roles of disability, fatigue, mood, pain, self-efficiency, and social support	292 adults with MS<br><br>Mean age:48 years	Actigraph acceleromet er<br><br>7 days	Total movement/time spent in activity.	FSS questionnair e	After running a full information maximum likelihood (FIML) estimation between physical activity and fatigue scores homed an indirect association between physical activity such that those who were more physical active reported low of fatigue.
Ana Jessica Pinto (2023) [67]	Increased prolonged sitting in patients with rheumatoid arthritis during the covid-19	64 PWRA<br>Mean age:61 years	Activ PAL micro<br>7 days	Measures of time spent siting and lying in prolonged sitting, standing	FSS questionnair e	Changes in physical activity and sedentary behavior levels were not associated with changes in fatigue by computing a person correlation coefficient between them.

	pandemic: a within-subjects accelerometer-based study.			,stepping in light intensity physical activity, MVPA and number of sit to stand times and time in sedentary behavior.		

The table above summarizes all the included studies in this review.

Supplementary Table 2| Search terms used.

String no.	Query
#1. (Disease)	chronic disease* /or multiple long-term condition* /or chronic illness* /or long-term dis* /or chronic sick* /or rheumatoid* /or multiple sclerosis /or long covid /or chronic heart failure /or Parkinson* .mp
#2 (Fatigue)	Fatigue /or exhaustion /or lassitude /or mental fatigue /or tired * .mp
#3 (Digital biomarkers)	Remote monitor* / or wearable* / or digital sensor* / or digital health tech* /or DHT /or digital biomarker* /or digital measure* / or imu* / or accelerometer* /or smart watch* /or smartwatch* / or activity tracker* /or sensor /or digital measurement*.mp

The table above states all the search terms used in this review.

Supplementary Table 3 | Certainty of Evidence

Biomarker Category	Specific Measure	Chronic Conditions	Number of Supporting Studies	Consistency	Certainty of Evidence
Physical Activity Metrics	Daily step count	MS, RA, COPD, Long COVID, Cancer	7 [13,17,18,19,29,36,39]	Consistent	High
Physical Activity Metrics	MVPA (minutes/day)	MS, RA, COPD, Long COVID, Cancer	9 [13,14,19,24,34,35,36,40,22]	Consistent	High
Physical Activity Metrics	Sedentary time (minutes/day)	MS, RA, COPD, Cancer	4 [14,29,31,39]	Consistent	High
Physical Activity Metrics	Activity counts per day	MS, RA, CFS	5 [14,15,20,41,42]	Consistent	High
Physical Activity Metrics	Sit-to-stand time (seconds)	RA	1 [30]	Not consistent	Low
Sleep Measures	Sleep efficiency/fragmentation	Cancer	2 [38,63]	Not consistent	Low
Autonomic Measures	HRV (Various metrics: RMSSD, HF, RR intervals)	MS, COPD, IBD, PSS, SLE	5 [17,18,45,46,36]	Consistent	High

Gait Parameters	Step time asymmetry	PD	1 [44]	Not consistent	Low
Gait Parameters	Gait speed (m/s)	PD	1 [44]	Not consistent	Low

Note: Consistency was defined as "consistent" if a biomarker was evaluated in three or more studies with similar findings; otherwise, it was considered "not consistent." Certainty of evidence was rated as "high" if the biomarker demonstrated consistency and the supporting studies were of high quality. If consistency was present but supporting studies were of moderate quality, certainty was rated as "moderate." If the biomarker was evaluated in fewer than three studies, or if findings were inconsistent, certainty of evidence was rated as "low," regardless of individual study quality.

The table above represents the certainty of evidence for each biomarker studied in this review.

Supplementary Table 4 – Prisma Checklist

Section and Topic	Item #	Checklist item	Location where item is reported
**TITLE**			
Title	1	Identify the report as a systematic review.	Title page
**ABSTRACT**			
Abstract	2	See the PRISMA 2020 for Abstracts checklist.	Abstract page
**INTRODUCTION**			
Rationale	3	Describe the rationale for the review in the context of existing knowledge.	Introduction page
Objectives	4	Provide an explicit statement of the objective(s) or question(s) the review addresses.	Introduction page
**METHODS**			
Eligibility criteria	5	Specify the inclusion and exclusion criteria for the review and how studies were grouped for the syntheses.	Materials and methods page
Information sources	6	Specify all databases, registers, websites, organisations, reference lists and other sources searched or consulted to identify studies. Specify the date when each source was last searched or consulted.	Materials and methods page
Search strategy	7	Present the full search strategies for all databases, registers and websites, including any filters and limits used.	Materials and methods page
Selection process	8	Specify the methods used to decide whether a study met the inclusion criteria of the review, including how many reviewers screened each record and each report retrieved, whether they worked independently, and if applicable, details of automation tools used in the process.	Materials and methods page
Data collection process	9	Specify the methods used to collect data from reports, including how many reviewers collected data from each report, whether they worked independently, any processes for obtaining or confirming data from study investigators, and if applicable, details of automation tools used in the process.	Materials and methods page

Section and Topic	Item #	Checklist item	Location where item is reported
Data items	10a	List and define all outcomes for which data were sought. Specify whether all results that were compatible with each outcome domain in each study were sought (e.g. for all measures, time points, analyses), and if not, the methods used to decide which results to collect.	Materials and methods page
	10b	List and define all other variables for which data were sought (e.g. participant and intervention characteristics, funding sources). Describe any assumptions made about any missing or unclear information.	Materials and methods page
Study risk of bias assessment	11	Specify the methods used to assess risk of bias in the included studies, including details of the tool(s) used, how many reviewers assessed each study and whether they worked independently, and if applicable, details of automation tools used in the process.	Materials and methods page
Effect measures	12	Specify for each outcome the effect measure(s) (e.g. risk ratio, mean difference) used in the synthesis or presentation of results.	Not Applicable
Synthesis methods	13a	Describe the processes used to decide which studies were eligible for each synthesis (e.g. tabulating the study intervention characteristics and comparing against the planned groups for each synthesis (item #5)).	Not applicable - no meta-analysis conducted
	13b	Describe any methods required to prepare the data for presentation or synthesis, such as handling of missing summary statistics, or data conversions.	Not applicable - no meta-analysis conducted
	13c	Describe any methods used to tabulate or visually display results of individual studies and syntheses.	Not applicable - no meta-analysis conducted
	13d	Describe any methods used to synthesize results and provide a rationale for the choice(s). If meta-analysis was performed, describe the model(s), method(s) to identify the presence and extent of statistical heterogeneity, and software package(s) used.	Not applicable - no meta-analysis conducted
	13e	Describe any methods used to explore possible causes of heterogeneity among study results (e.g. subgroup analysis, meta-regression).	Not applicable -

Section and Topic	Item #	Checklist item	Location where item is reported
			no meta-analysis conducted
	13f	Describe any sensitivity analyses conducted to assess robustness of the synthesized results.	Not applicable - no meta-analysis conducted
Reporting bias assessment	14	Describe any methods used to assess risk of bias due to missing results in a synthesis (arising from reporting biases).	Not applicable - no meta-analysis conducted
Certainty assessment	15	Describe any methods used to assess certainty (or confidence) in the body of evidence for an outcome.	Methods and Results sections
**RESULTS**			
Study selection	16a	Describe the results of the search and selection process, from the number of records identified in the search to the number of studies included in the review, ideally using a flow diagram.	Results page
	16b	Cite studies that might appear to meet the inclusion criteria, but which were excluded, and explain why they were excluded.	Results page
Study characteristics	17	Cite each included study and present its characteristics.	Results page
Risk of bias in studies	18	Present assessments of risk of bias for each included study.	Results page
Results of individual studies	19	For all outcomes, present, for each study: (a) summary statistics for each group (where appropriate) and (b) an effect estimate and its precision (e.g. confidence/credible interval), ideally using structured tables or plots.	Results page
Results of syntheses	20a	For each synthesis, briefly summarize the characteristics and risk of bias among contributing studies.	Not applicable
	20b	Present results of all statistical syntheses conducted. If meta-analysis was done, present for each the summary estimate and its precision (e.g. confidence/credible interval) and measures of statistical heterogeneity. If comparing groups, describe the direction of the effect.	Not applicable
	20c	Present results of all investigations of possible causes of heterogeneity among study results.	Not applicable

Section and Topic	Item #	Checklist item	Location where item is reported
	20d	Present results of all sensitivity analyses conducted to assess the robustness of the synthesized results.	Not applicable
Reporting biases	21	Present assessments of risk of bias due to missing results (arising from reporting biases) for each synthesis assessed.	Results section
Certainty of evidence	22	Present assessments of certainty (or confidence) in the body of evidence for each outcome assessed.	Results section
**DISCUSSION**			
Discussion	23a	Provide a general interpretation of the results in the context of other evidence.	Available on discussion page
	23b	Discuss any limitations of the evidence included in the review.	Available on discussion page
	23c	Discuss any limitations of the review processes used.	Available on discussion page
	23d	Discuss implications of the results for practice, policy, and future research.	Available on discussion page
**OTHER INFORMATION**			
Registration and protocol	24a	Provide registration information for the review, including register name and registration number, or state that the review was not registered.	Available on materials and methods page
	24b	Indicate where the review protocol can be accessed, or state that a protocol was not prepared.	Available on materials and methods page

Section and Topic	Item #	Checklist item	Location where item is reported
	24c	Describe and explain any amendments to information provided at registration or in the protocol.	Available on materials and methods page
Support	25	Describe sources of financial or non-financial support for the review, and the role of the funders or sponsors in the review.	Available on page 2
Competing interests	26	Declare any competing interests of review authors.	Available on page 2
Availability of data, code and other materials	27	Report which of the following are publicly available and where they can be found: template data collection forms; data extracted from included studies; data used for all analyses; analytic code; any other materials used in the review.	Available on materials and methods page

From: Page MJ, McKenzie JE, Bossuyt PM, Boutron I, Hoffmann TC, Mulrow CD, et al. The PRISMA 2020 statement: an updated guideline for reporting systematic reviews. BMJ 2021;372:n71. doi: 10.1136/bmj.n71. This work is licensed under CC BY 4.0. To view a copy of this license, visit https://creativecommons.org/licenses/by/4.0/

The table above is the prisma checklist
